# Focusing the diversity of *Gardnerella vaginalis* through the lens of ecotypes

**DOI:** 10.1111/eva.12555

**Published:** 2017-11-16

**Authors:** Omar E. Cornejo, Roxana J. Hickey, Haruo Suzuki, Larry J. Forney

**Affiliations:** ^1^ School of Biological Sciences Washington State University Pullman WA USA; ^2^ Department of Biological Sciences University of Idaho Moscow ID USA; ^3^ Institute for Bioinformatics and Evolutionary Studies University of Idaho Moscow ID USA; ^4^ Institute for Advanced Biosciences Keio University Tsuruoka Japan; ^5^ Present address: Phylagen Inc. San Francisco CA USA

**Keywords:** bacterial vaginosis, *Gardnerella vaginalis*, genetic diversity, genomes

## Abstract

*Gardnerella vaginalis* has long been associated with bacterial vaginosis, a condition that increases the risk of women to preterm birth, sexually transmitted infections, and other adverse sequelae. However, *G. vaginalis* is also commonly found in healthy asymptomatic women of all ages. This raises the question if genetic differences among strains might distinguish potentially pathogenic from commensal strains. To disentangle the diversity of *G. vaginalis,* we invoked the concept of ecotypes—lineages of genetically and ecologically distinct strains within a named species—to better understand their evolutionary history and identify functional characteristics. We compared the genomes of *G. vaginalis* to six species in the closely related *Bifidobacterium* genus and found that *G. vaginalis* has a large accessory genome relative to *Bifidobacterium*, including many unique genes possibly involved in metabolism, drug resistance, and virulence. We then performed a comparative genomic analysis of 35 strains of *G. vaginalis* to infer a phylogeny based on the combined analysis of the core genome, using nucleotide substitution models, and the accessory genome, using gene gain/loss models. With the inferred tree topology, we performed comparisons of functional gene content among lineages that diverged at varying depths in the phylogeny and found significant differences in the representation of genes putatively involved in pathogenicity. Our functional enrichment analysis suggests that some lineages of *G. vaginalis* may possess enhanced pathogenic capabilities, including genes involved in mucus degradation like sialidases, while others may be commensal strains, lacking many of these pathogenic capabilities. The combined phylogenetic evidence and functional enrichment analysis allowed us to identify distinct ecotypes that have evolved in *G. vaginalis* as the result of the differential gene gain/loss for specific functions, including the capability to cause disease. We finally discuss how this analysis framework could be used to gain insight into the etiology of bacterial vaginosis and improve diagnosis.

## 1 INTRODUCTION

Bacteria in the human vagina are known to play a significant role in urogenital health, but it is not always clear whether particular species or strains are beneficial or detrimental to health. *Gardnerella vaginalis*, perhaps more than any other species in the vaginal ecosystem, has been scrutinized because of its close association with bacterial vaginosis (BV) (Koumans et al., 2007). The connection between *G. vaginalis* and BV dates back to 1955, when Gardner and Dukes first classified the small, Gram‐positive (though variable staining), pleomorphic rods as *Haemophilus vaginalis* (Criswell, Marston, Stenback, Black, & Gardner, 1971; Gardner & Dukes, 1955). It was later reclassified as *Corynebacterium vaginale* (Zinnemann & Turner, 1963) before eventually being renamed after its discoverer as *Gardnerella vaginalis* (Greenwood & Pickett, 1980). Today *G. vaginalis* remains the only recognized species in its genus, with its closest relatives found in the genus *Bifidobacterium*. Postulated virulence mechanisms of *G. vaginalis* include biofilm formation (Patterson, Stull‐Lane, Girerd, & Jefferson, 2010; Swidsinski et al., 2005; Verstraelen & Swidsinski, 2013), secretion of an exotoxin (vaginolysin) (Cauci et al., 1993; Gelber, Aguilar, Lewis, & Ratner, 2008), and the production of enzymes that enable degradation of vaginal mucus (Gilbert, Lewis, & Lewis, 2013; Wiggins, Hicks, Soothill, Millar, & Corfield, 2001). The prevalence of *G. vaginalis* in BV approaches 100% (Bradshaw et al., 2006; Srinivasan et al., 2012; Verhelst et al., 2004; Zariffard, Saifuddin, Sha, & Spear, 2002), and although other bacteria have been implicated in the etiology of BV, *G. vaginalis* is often considered a primary indicator of the disease (Muzny & Schwebke, 2013).

The strong correlation between BV and *Gardnerella* has sometimes been taken as direct evidence of causation (Schwebke, Muzny, & Josey, 2014b). Nevertheless, there are many instances in which *G. vaginalis* is present but the symptoms of BV are not. *G. vaginalis* is often a major constituent of the vaginal microbiota of healthy, asymptomatic women of all ages (Fredricks, Fiedler, & Marrazzo, 2005; Ravel et al., 2011; Schwebke, Flynn, & Rivers, 2014a) including young girls (Hickey et al., 2015) and postmenopausal women (Shen et al., 2016). Studies have shown that *G. vaginalis* can be a prominent member of vaginal communities in upwards of 40% of healthy individuals (Aroutcheva, Simoes, Behbakht, & Faro, 2001; Tabrizi, Fairley, Bradshaw, & Garland, 2006) and Balashov, Mordechai, Adelson, and Gygax (2014) found *G. vaginalis* in 97% of asymptomatic subjects with bacterial vaginosis using qPCR. The common occurrence of a putative pathogen in asymptomatic women is a paradox that needs resolution so that the role of *G. vaginalis* in BV pathogenesis can be properly understood (Catlin, 1992). One possibility is that only certain lineages of *G. vaginalis* are pathogenic and others are natural commensals. In accordance with this notion, researchers have grappled with delineating “good” and “bad” strains of *Gardnerella* using a variety of techniques that characterize within‐species diversity by phenotypic or genotypic profiling (Ingianni, Petruzzelli, Morandotti, & Pompei, 1997; Jayaprakash, Schellenberg, & Hill, 2012; Piot et al., 1984). More recently, comparative genomics studies have revealed substantial differences in gene composition that surpass even some of the most diverse species known (Ahmed et al., 2012; Harwich et al., 2010; Yeoman et al., 2010). Some studies suggest particular biotypes or genotypes display a greater association with BV (Benito, Vazquez, Berron, Fenoll, & Saez‐Neito, 1986; Numanović et al., 2008), but results are inconsistent (Aroutcheva et al., 2001; Piot et al., 1984) and may be confounded by erroneous biotype identification (Moncla & Pryke, 2009) or the presence of multiple types of *G. vaginalis* within a single individual (Balashov et al., 2014; Briselden & Hillier, 1990; Santiago et al., 2011). It is then important to understand the functional basis of diversification in *G. vaginalis* that could impact how lineages interact with the host and other microbes, and not just genetic differentiation resulting from natural demographic processes. Functional studies have shown that *G. vaginalis* has a high ability to adhere to epithelial cells and presents increased cytotoxicity when compared to other bacteria associated with BV (Patterson et al., 2010). Recent functional studies have shown that BV isolates of *G. vaginalis* present higher cytotoxicity than non‐BV isolates (Castro et al., 2015), and no differences in the ability to establish in the presence of competitors (*Lactobacillus crispatus*) were found between BV and non‐BV isolates (Castro et al., 2015). These results and single genome comparisons between a BV strain and a non‐BV strain show no difference in cytolysin proteins encoded and yet lower cytotoxicity in non‐BV strains (Harwich et al., 2010) make it clear that additional work is needed to identify relevant functional characteristics between pathogenic and nonpathogenic strains.

Here, we employ the concept of bacterial ecotypes (Cohan, 2001, 2006) to disentangle the diversity of bacterial lineages and use *G. vaginalis* as a model to illustrate how it can be classified into ecologically meaningful and clinically useful entities. An ecotype is defined as a set of strains that are genetically similar to one another but ecologically distinct from others (Cohan, 2001). Genetic similarity (or “cohesion”) is characterized using a phylogenetic approach to identify sequence clusters that reflect shared evolutionary history. Ecological distinctness can be inferred by determining sets of shared genes or similarities in gene expression patterns under the same environmental conditions. Ecotypes thus represent lineages within a species that possess unique adaptations and ecological capabilities. In this work, we performed phylogenetic and functional analyses to determine whether evolutionary relationships inferred from the genome sequences of 35 *G. vaginalis* isolates were associated with ecological differences inferred from differences in their gene repertoire. Our findings provide support for the separation of *G. vaginalis* into multiple ecotypes that have distinctive phylogenetic signals and functional genes. This information could be used to more accurately identify and characterize types of *G. vaginalis* associated with symptoms of bacterial vaginosis, ultimately improving diagnostic procedures for the disease.

## 2 MATERIALS AND METHODS

### 2.1 Genome sequences

We downloaded 35 genome sequences (three complete and 32 draft) of *Gardnerella vaginalis* isolates from the PATRIC database (Table 1, genome data archived at ftp://ftp.patricbrc.org/patric2/) (Wattam et al., 2014). Although 36 strains were available at the time of analysis, we excluded strain 6420LIT because it was incompletely sequenced. We downloaded DNA and amino acid sequences of the PATRIC coding DNA sequences (CDS) in FASTA format (file extensions *.PATRIC.ffn and *.PATRIC.faa, respectively, where * represents the strain name), along with relevant tables describing the protein annotations (*.PATRIC.cds.tab, *.PATRIC.features.tab). We also gathered functional annotations directly from PATRIC, including Gene Ontology (GO) function (*.PATRIC.go) and KEGG biochemical pathway assignments (*.PATRIC.path). Counting all 35 genomes, our initial data set included 44,505 protein CDS, 375 unique GO term annotations covering 14,896 CDS, and 121 unique pathway annotations covering 10,086 CDS.

**Table 1:** Genomic characteristics of *Gardnerella vaginalis* strains

Strain	GenBank accession	Genomic characteristics^a^					Source
		Size (Mb)	Contigs	Plasmids	GC%	CDS	
00703Bmash	ADET00000000	1.566	16	0	42.3	1,273	Vagina
00703C2mash	ADEU00000000	1.547	22	0	42.3	1,237	Vagina
00703Dmash	ADEV00000000	1.491	11	0	43.4	1,172	Vagina
0288E	ADEN00000000	1.709	17	0	41.2	1,364	Endometrium
101	AEJD00000000	1.527	43	0	43.4	1,190	NR
1400E	ADER00000000	1.716	28	0	41.2	1,370	Vagina/endometrium^b^
1500E	ADES00000000	1.548	27	0	43	1,195	Vagina/endometrium^b^
284V	ADEL00000000	1.651	16	0	41.2	1,304	Endometrium
315‐A	AFDI00000000	1.653	13	0	41.4	1,320	Vagina
409‐05	CP001849	1.618	1	0	42	1,190	Vagina
41V	AEJE00000000	1.659	76	0	41.3	1,336	Vagina
5‐1	ADAN00000000	1.673	94	0	42	1,294	Vagina
55152	ADEQ00000000	1.643	25	0	41.3	1,322	Vagina/endometrium^b^
6119V5	ADEW00000000	1.500	12	0	43.3	1,187	Vagina
6420B	ADEP00000000	1.494	14	0	42.2	1,162	Vagina/endometrium^b^
75712	ADEM00000000	1.673	3	0	41.3	1,314	Vagina
AMD	ADAM00000000	1.607	117	0	42.1	1,217	Vagina
ATCC 14018	ADNB00000000	1.604	145	0	41.2	1,313	NR
ATCC 14019	CP002104	1.667	1	0	41.4	1,345	Vagina
HMP9231	CP002725	1.727	1	0	41.2	1,376	Endometrium
JCP7275	ATJS00000000	1.560	202	0	41	1,230	Vagina
JCP7276	ATJR00000000	1.656	179	0	41	1,315	Vagina
JCP7659	ATJQ00000000	1.533	214	0	41.9	1,251	Vagina
JCP7672	ATJP00000000	1.601	169	0	41.2	1,251	Vagina
JCP7719	ATJO00000000	1.559	185	0	42	1,302	Vagina
JCP8017A	ATJN00000000	1.606	187	0	42.1	1,343	Vagina
JCP8017B	ATJM00000000	1.599	187	0	42	1,335	Vagina
JCP8066	ATJL00000000	1.515	197	0	42.2	1,209	Vagina
JCP8070	ATJK00000000	1.476	173	0	42.2	1,208	Vagina
JCP8108	ATJJ00000000	1.663	176	0	41.1	1,351	Vagina
JCP8151A	ATJI00000000	1.556	189	0	42	1,259	Vagina
JCP8151B	ATJH00000000	1.551	185	0	42.2	1,276	Vagina
JCP8481A	ATJG00000000	1.567	204	0	42.9	1,263	Vagina
JCP8481B	ATJF00000000	1.570	180	0	42.9	1,251	Vagina
JCP8522	ATJE00000000	1.470	191	0	42.2	1,180	Vagina

a Genomes were downloaded from the PATRIC database in February 2015 (ftp://ftp.patricbrc.org/patric2/). CDS = coding DNA sequence.

b PATRIC metadata differs from report by Ahmed et al. (2012).

We also selected 20 strains of *Bifidobacterium* spp. (Table S1), the most closely related genus to *Gardnerella*, to compare genomic differences between the two genera. To narrow our selection, we focused on strains that were isolated from a human, selected complete over draft genome sequences if available and ignored any strains lacking PATRIC CDS features. The same file types described above were downloaded from PATRIC (ftp://ftp.patricbrc.org/patric2/). Counting all 20 genomes, the initial data set included 38,276 protein CDS, 537 unique GO term annotations covering 11,999 CDS, and 130 unique pathway annotations covering 8,488 CDS.

### 2.2 Software and bioinformatic analysis

We employed several bioinformatic software programs in our computational analyses, including BLASTP (Altschul et al., 1997), ClustalW (Larkin et al., 2007), MUSCLE (Edgar, 2004), OrthoMCL (v2.0.9) (Li, Stoeckert, & Roos, 2003), and RAxML (v8.0.3) (Stamatakis, 2014). Downstream analysis and graphical summarization was performed in R (v3.1.0) using packages Biostrings (Pagès et al., 2016), ape (Paradis, Claude, & Strimmer, 2004), and vegan (Oksanen, Blanchet, Kindt, & Legendre, 2013). These analyses are described in greater depth below.

### 2.3 Maximum‐likelihood phylogenetic analysis of 16S rRNA genes

We downloaded DNA sequences of RNA coding genes from the PATRIC database (file extensions *.PATRIC.frn; archived at ftp://ftp.patricbrc.org/patric2/) for all *G. vaginalis* and *Bifidobacterium* genomes. 16S rRNA genes were distinguished by being annotated as “Small Subunit Ribosomal RNA” (ssuRNA). We considered sequences >1,400 bp to be full‐length genes and retained them for further analysis; these were available for 18 *G. vaginalis* genomes and all 20 *Bifidobacterium* genomes. Several genomes possessed multiple gene copies, and we removed any within‐strain exact sequence duplicates prior to phylogenetic analysis. We performed multiple sequence alignment in MUSCLE (Edgar, 2004) and computed the maximum‐likelihood phylogeny in RAxML (Stamatakis, 2014) under the generalized time‐reversible model of substitution rates drawn from a gamma distribution with 1,000 bootstrap replicates.

### 2.4 Total‐evidence maximum‐likelihood phylogenetic analysis of core and accessory genes

In addition to the concatenated alignment from the core set of genes previously identified for all *G. vaginalis*, we prepared a comprehensive table with the presence and absence of the accessory genes (i.e., not common to all 35 accessions of *G. vaginalis*). Sites with gaps were removed from the analysis. With the combined set, we inferred a maximum‐likelihood phylogeny while considering a substitution model of evolution for the DNA alignment and a stepwise model with simple transitions to explain the gain/loss of a gene. We performed our analysis using a generalized time‐reversible model of DNA substitution that considered heterogeneity in substitution rates (gamma distribution with four categories), and parameters independently fitted to first, second, and third positions of the codons. Our stepwise model of evolution was a simple gain/loss transition matrix, proposed by Lewis (2001) to analyze discrete morphological data, to model the evolution of gene presence/absence along the tree. The analyses were performed in RAxML (Stamatakis, 2014), and support for the clades was calculated from 1,000 bootstrap replicates. A majority‐rule criterion was used to compute the consensus tree.

### 2.5 Identification of homologous protein families

In the analysis of 35 *G. vaginalis* genomes, we performed an all‐against‐all similarity analysis on the amino acid sequences of 44,505 CDS using BLASTP (Altschul et al., 1997) with an *E*‐value cutoff of 1E−10 to construct a database of homologous protein sequences. We then used OrthoMCL to cluster them into protein families (sets of orthologous and paralogous protein encoding genes) specifying a minimum 70% identity threshold and *E*‐value cutoff of 1E−5. We employed the same approach for the analysis of both *G. vaginalis* and *Bifidobacterium* spp. genomes (total of 82,781 CDS among 55 genomes) except that we relaxed the minimum identity threshold to 50% to allow for greater divergence in protein families among genera. Due to differences in the definitions of homologs (i.e., genes evolved from a common ancestral sequence), orthologs (i.e., genes evolved from a common ancestor and separated by speciation), and paralogs (i.e., genes related by duplication within a genome), we agnostically refer to clusters of protein CDS as “protein families” (or simply genes when we are referring to the DNA sequences). Protein families and singletons were summarized in tables, and data were output as binary values (indicating the presence or absence of each protein family in each genome) and counts (indicating the number of CDS assigned to each protein family in each genome).

### 2.6 Identification of core, accessory, and unique protein families

We used the OrthoMCL binary tables to determine which protein families were shared among all genomes (i.e., core proteins), shared among only some genomes (i.e., accessory proteins), or unique to individual genomes. We performed similar analyses on all genomes together as well as for the nested groups of genomes determined by the topology of the inferred phylogeny (described below). We also estimated pan‐genome size with protein family accumulation curves using the package vegan in R (v2.2‐1) (Oksanen et al., 2013). We built two different models of accumulation, a log model (*cg* = *b *+ *a* * log(ng)) and a power model (*cg* = *b* * ng^*a*^), where cg is gene content (total number of new genes), ng is the number of genomes observed, and *a* and *b* are parameters of the model. The log model is a model that saturates as the number of genomes increases as the derivative of the function evaluated in the limit is zero. The power model does not converge to zero as the number of genomes observed increases and better represents an open pan‐genome.

### 2.7 Protein family enrichment analysis

We performed statistical analyses to evaluate protein family and functional category presence, absence, and relative abundance among the subgroups or clades of *G. vaginalis* that had independent most recent common ancestors. This was performed for several annotated data sets to identify specific functional annotations, Gene Ontology (GO) categories, and KEGG biochemical pathways that distinguished nested groups from one another. For each comparison, we constructed a two‐by‐two contingency table for each GO functional category, KEGG pathway, or protein family (as determined by OrthoMCL clustering). Each table included the following parameters: the number of group genomes’ protein CDS present in this category (*a*); the number of group genomes’ CDS not in this category (*b*); the number of other genomes’ CDS in this category (*c*); and the number of other genomes’ CDS not in this category (*d*). We used the odds ratio (defined as *ad*/*bc*) to rank the relative overrepresentation (odds ratio >1) or underrepresentation (odds ratio <1) of each functional category. Finally, to account for multiple comparisons we adjusted *p‐*values obtained by Fisher's exact test by controlling for the false‐discovery rate (Benjamini & Hochberg, 1995) and reported these as *q‐*values.

## 3 RESULTS

### 3.1 16S rRNA phylogeny of *Gardnerella vaginalis* and selected *Bifidobacterium* spp.

The overall goal of this study was to identify putative ecotypes using the phylogeny of *G. vaginalis* strains as a framework to identify and describe functional differences among related groups. For this purpose, we selected 35 *G. vaginalis* strains with whole‐genome sequences available in the PATRIC database (http://patricbrc.org) that were reportedly isolated from the human vagina or endometrium (Table 1). Furthermore, to better understand how *Gardnerella* has diverged from *Bifidobacterium* over evolutionary time, we chose 20 strains of six bifidobacteria species (Table S1) that were reportedly isolated from the human gastrointestinal tract (*n* = 15), urogenital tract (*n* = 2), or mammary gland (*n* = 1); the body site of origin was not reported for two *B. animalis* strains.

**Figure 1:**
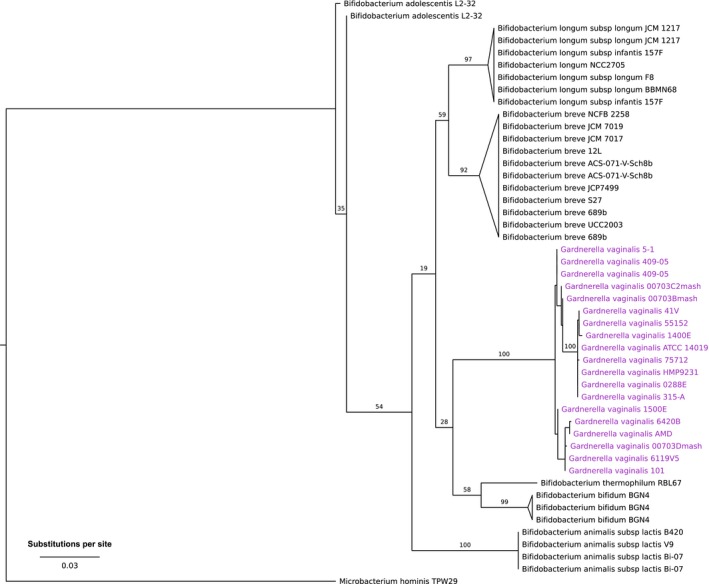
Maximum‐likelihood 16S rRNA gene phylogeny of 18 strains of *G. vaginalis* and 20 strains of *Bifidobacterium* spp. The maximum‐likelihood phylogeny was computed on aligned DNA sequences of 16S rRNA genes (>1,400 bp) under the GTR + gamma model of sequence evolution with 1,000 bootstrap replicates. Bootstrap support values are indicated on the branches. Terminal branches were collapsed by species except for *G. vaginalis*. Some strains possess multiple distinct gene copies and are represented multiples times on the tree

The 16S rRNA phylogeny of 18 *G. vaginalis* and 20 *Bifidobacterium* strains with available full‐length ribosomal gene sequences is shown in Figure 1. *G. vaginalis* forms a monophyletic group nested within the *Bifidobacterium* genus, and its closest relatives appear to be *B. bifidum* and *B. thermophilum*. The *G. vaginalis* strains themselves were highly similar in terms of 16S rRNA sequence, with pairwise similarity ranging from 98.7% to 100.0%. In contrast, the pairwise similarities of *G. vaginalis* strains to the 20 *Bifidobacterium* strains ranged from 91.5% to 94.8%. Members of the two genera were strikingly different in both genome size and GC content. Genomes of *G. vaginalis* (Table 1) ranged in size from 1.47 to 1.73 Mb (average 1.59 Mb) and varied in GC content from 41.0% to 43.4% (average 41.9%). In contrast, the genomes of *Bifidobacterium* species (Table S1) ranged from 1.94 to 2.42 Mb (average 2.27 Mb) with a GC content of 58.6%–62.6% (average 59.6%), which was consistent with many other *Bifidobacterium* spp. that have been previously studied (Bottacini et al., 2010; Lukjancenko, Ussery, & Wassenaar, 2011). These data suggest the genomes of *Gardnerella* have undergone a substantial reduction in size after diverging from their *Bifidobacterium* relatives. This is striking considering their 16S rRNA gene sequences are >91% identical, a level that would typically place bacterial taxa in the same genus (Janda & Abbott, 2007).

### 3.2 Pan‐genome of *Gardnerella vaginalis*

To define the pan‐genome of *G. vaginalis*, we clustered 44,505 coding DNA sequences (CDS) into protein families using an algorithm that groups orthologous sequences with >70% amino acid sequence identity. Sequences that could not be grouped with any others at this threshold were deemed singletons. We further annotated the clusters of orthologous genes as belonging to known protein families using KEGG. The resulting pan‐genome of *Gardnerella* consisted of 2,392 protein families that were present in two or more strains, 7 protein families that were present in only one strain (i.e., two or more CDS from a single strain that clustered into one protein family), and 1,495 singletons. Of the 2,399 protein families found, 49.4% were annotated as hypothetical proteins, so the functional attributes of a large portion of *G. vaginalis* genomes are unknown. To further characterize the pan‐genome of *G. vaginalis*, we fitted two contrasting models to the accumulation curve (Fig. S1) and found that a power model better explains the accumulation of new genes the number of genomes considered increases, suggesting that the accessory genome is unbounded and increases as more genomes are included in the analysis. Partitioning the pan‐genome into core and accessory components revealed a small core genome and an expansive accessory genome (Fig. S2). The core genome of all 35 strains included 694 protein families, which was just 29.0% of the total 2,392 protein families that were shared by two or more strains. The remaining protein families constituted a large accessory genome in which many genes were present in only one or a few strains. Furthermore, each strain had several unique genes (singletons) that were not homologous to any others at a 70% amino acid identity threshold.

### 3.3 Comparison of *Gardnerella* and *Bifidobacterium* spp. pan‐genomes

To understand the functional potential of populations within the genus *Gardnerella*, we compared the protein‐coding genomes of 35 *G. vaginalis* and 20 *Bifidobacterium* strains to estimate the collective pan‐genome. Using a relaxed threshold of 50% amino acid identity to allow for greater divergence in protein families between genera, we identified 4,633 homologous protein families among the 82,781 CDS present in the two genera. *G. vaginalis* strains shared 703 core protein families, while *Bifidobacterium* strains shared 906 core protein families. Thus, even though *G. vaginalis* is considered a single species, it has fewer protein families common to all of its members than do a collection of six species of *Bifidobacterium*. The core genomes of *Gardnerella* and *Bifidobacterium* had 553 protein families in common, representing 78.6% of the core genome of *Gardnerella* and 61.0% of the core genome of *Bifidobacterium*. We found a relatively large accessory genome in *G. vaginalis* of 1,720 protein families, which was comparable in number to the accessory genome of all strains from six species of *Bifidobacterium* (*n* = 1,757 protein families). These data reveal that the within‐species differences in genomic content among *G. vaginalis* strains are on par or even greater than those observed among multiple species of *Bifidobacterium*. This comparison sets the framework to compare the protein and functional capabilities of *Gardnerella* and *Bifidobacterium*, as well as among different lineages of *G. vaginalis*.

### 3.4 Gene set enrichment analysis of *G. vaginalis versus Bifidobacterium* spp.

To gain insight into the functional differences between *Gardnerella* and *Bifidobacterium*, we performed a protein family enrichment analysis on the protein families, GO categories, and biochemical pathways that were represented in the genomes of the two genera. After adjusting for multiple comparisons, we identified 785 protein families that were found in *Bifidobacterium* genomes but absent from *Gardnerella*, 30 underrepresented in *Gardnerella*, 88 overrepresented in *Gardnerella*, and 282 families unique to *Gardnerella* (File S1). Among the protein families unique to *Gardnerella* were several putative virulence factors including zeta toxin, YafQ toxin, other toxin–antitoxin proteins, and thiol‐activated cytolysin. Additionally, several protein families were annotated as transporter proteins, which may be used to acquire nutrients that *Gardnerella* is unable to synthesize. Given their smaller genomes, it is not surprising that *G. vaginalis* also had fewer GO categories and biochemical pathways than species of *Bifidobacterium*. The results show that beta‐galactosidase, alpha‐galactosidase, and alpha‐glucosidase were significantly less prevalent in *G. vaginalis* relative to species of *Bifidobacterium*, probably because only strains in clade/ecotype 1 (described below) possessed such genes. However, the exo‐alpha‐sialidase GO category was significantly overrepresented in *G. vaginalis* (OR = 3.59, *q *=* *7.88E−04) and only 10 of 20 *Bifidobacterium* strains possessed genes encoding sialidase (all were *B. bifidum* or *B. breve*). Collectively, these findings suggest that while *G. vaginalis* lacks many of the metabolic capabilities present in *Bifidobacterium* spp., it appears to encode many proteins that may confer greater resistance to antibiotics, kill other bacteria via toxin–antitoxin systems, or enhance degradation of vaginal mucus by sialidase.

### 3.5 Phylogenetic and gene enrichment analysis to identify putative ecotypes of *G. vaginalis*

Ecotypes should both possess distinguishing ecological characteristics and exhibit genetic cohesiveness as evident from clustering of gene sequences among strains. By combining these two criteria, one can assess whether ecologically similar strains were derived from a common ancestor or arose through the convergent evolution of adaptive traits, or a combination of both (Cohan, 2002). In this regard, a group of lineages with a most recent common ancestor in a phylogeny defines a clade (at any given level in the hierarchy of a phylogeny); but for isolates in a clade to belong to an ecotype, they have to present functional distinctiveness. We used the data on genome divergence and gene composition in a two‐step process to identify relevant functional differences among clades that would result in these being considered ecotypes.

**Figure 2:**
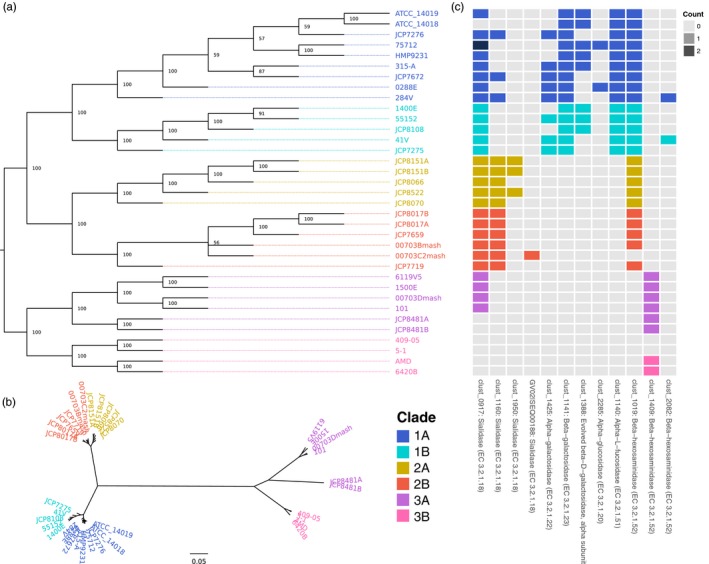
Majority‐rule consensus tree estimated on the concatenated sequence of the core genome (664 single‐copy genes) and the presence/absence data of accessory genes. (a) Unscaled tree; branch labels indicate bootstrap support (as percentage of gene trees that contain a bipartition). Tips are labeled with the *G. vaginalis* strain identifiers and colored according to putative taxonomic clades. (b) Majority‐rule consensus tree with topology fixed as shown in (a) with branch lengths scaled by the average core gene distances. (c) Counts of select protein families with putative mucus degradation capability that were differentially enriched among clades of *G. vaginalis*. The most prevalent annotation among each protein family is listed to along with the OrthoMCL cluster identifier. The clade color scheme in (b) applies to all panels of the figure

The first step involved inferring the phylogenetic of *G vaginalis* strains. For this, we integrated for the first‐time information from the core and accessory genomes to fit a nucleotide substitution model on the core genome and a gene gain/loss model of accessory genes to improve the resolution and support to nodes in the phylogeny. The inferred phylogeny (Figure 2a) was used in a second step as a framework to evaluate the differential enrichment of genes belonging to specific functional categories at different depths in the phylogeny. We progressively tested whether independent groups of lineages presented significant enrichment or loss of genes associated with a function or group of functions. This second step allowed us to define ecotypes: groups of strains with ecologically relevant differentiation via functional specialization. Because this tree was built on *G. vaginalis* alone, we used the phylogenetic relationships inferred from 16S rRNA gene sequences (Figure 1) to root the phylogenetic tree.

Bootstrap support for the internal nodes (Figure 2a) shows that we could confidently assign relationships among isolates inside previously defined clades. We also used the inferred topology to estimate evolutionary distances among lineages by fitting a generalized time‐reversible substitution model to the core genes. From this, we found three distinct clades that were highly divergent (designated as clades 1, 2, and 3 in Figure 2a,b). We observed longer average distances among lineages in clade 3 compared to clades 1 and 2, which could be the result of accelerated rates of evolution or insufficient sampling within the clade (Figure 2b). Our approach allowed us to resolve the internal structure of the phylogenetic clusters, a main difference between our approach and those published previously (Ahmed et al., 2012; Schellenberg, Patterson, & Hill, 2017). Our results suggest that, as more genomes are analyzed, there is a possibility that additional clades of interest will be defined.

In what follows, we used the phylogenetic relationships among *G. vaginalis* strains as an evolutionary framework to identify ecotypes that would correspond to clades with different metabolic or functional capabilities. For this, we compared the number of genes per functional category between groups of lineages with independent most recent common ancestors (i.e., separate clades in the phylogeny with no shared paths) and assessed whether there was a significantly different representation of functional capabilities between groups. This procedure ensured that all comparisons were independent from each other, conceptually following the same principle as the phylogenetic independent contrasts (Felsenstein, 1985). Comprehensive results are available in Files S2 and S3, and we highlight relevant results below. We observed a large number of significantly enriched functional genes, GO categories, and biochemical pathways across three major clades of strains. These data support the hypothesis that three genetically and ecologically distinct lineages of *G. vaginalis* exist and possess significant differences in their protein repertoire.

### 3.6 Enrichment of pathways and protein families in ecotype 3 and combined ecotypes 1/2

To define ecotypes, we first explored the phylogeny from the root to the tips and selected the first bifurcation corresponding to the separation of clade 3 from the combined clades 1 and 2 to perform enrichment analyses and identify the functional characteristics that define ecotype 3 and the combined ecotype 1/2. With an average genome size of 1.56 Mb, the ten isolates in clade 3 were more distinct from clades 1/2 in both phylogenetic distance and differential gene enrichment. Our enrichment analysis showed clear differentiation in the protein repertoires of ecotype 3 and combined ecotype 1/2. Ecotype 3 strains collectively had 51 unique, four overrepresented, seven underrepresented, and 47 absent protein families when compared to ecotypes 1/2 combined. We also found 13 unique, one overrepresented, five underrepresented, and nine absent GO categories in ecotype 3. Finally, we identified 10 underrepresented and four absent biochemical pathways in ecotype 3. While ecotype 3 is perhaps most notable for what it lacks relative to the other two clades, it does possess some unique features that may be interesting targets for future study. One protein family annotated as “membrane proteins related to metalloendopeptidases” was far more abundant in this group (OR = 13.36, *q* = 2.87E−03). Among the GO categories unique to this clade, two were associated with choline metabolism: choline kinase activity and choline‐phosphate cytidylyltransferase activity (*q* = 1.88E−04 in both cases). Notably, six strains within this clade (6119V5, 1500E, 00703Dmash, 101, JCP8481A, and JCP8481B) lack any protein families annotated as sialidase enzymes.

Perhaps the most striking overrepresented GO category was that of exo‐alpha‐sialidase activity in combined clades 1/2 (OR = 4.16, *q* = 2.43E−02). All isolates in this cluster possessed at least two distinct protein families annotated as sialidase; one in particular (clust_1160) was conserved among all 11 genomes of clade 2 (Figure 2c). While it is known that not all strains of *G. vaginalis* produce sialidase (Santiago et al., 2011), allelic diversity and gene copy numbers of sialidase have not been well characterized. Members of clade 2 lacked enzymes assigned to the GO category for thiamine biosynthesis; however, it had three unique protein families annotated as the three components of an energy‐coupling factor (ECF) transporter. ECF transporters are required for uptake of some micronutrients, with known substrates including riboflavin, thiamine, biotin, and tryptophan (Eitinger, Rodionov, Grote, & Schneider, 2011; Rodionov et al., 2009), suggesting members of clade 2 may obtain thiamine or other essential micronutrients from external sources.

### 3.7 Enrichment of pathways and protein families and differentiation of ecotypes 1 and 2

We further explored the phylogeny from the most recent common ancestor to all lineages in the combined ecotype 1/2 and took the first bifurcation corresponding to the separation of clade 1 from clade 2 identify the functional characteristics that define ecotypes 1 and 2. Ecotype 1, consisting of 14 strains with an average genome size of 1.66 Mb, had many significantly differentially represented protein families, GO categories, and biochemical pathways. Relative to the 11 strains from ecotype 2, genomes in this ecotype collectively possessed 46 unique, nine overrepresented, three underrepresented, and eight absent protein families; 10 unique, one overrepresented, and five absent GO categories; and three unique and two overrepresented biochemical pathways. Ecotype 2, with 11 genomes and an average genome size of 1.54 Mb, had relatively fewer significantly differentially enriched and unique protein families and GO categories. The pathway for galactose metabolism was enriched in ecotype 1 nearly two‐fold compared to ecotype 2 (OR = 1.63, *q* = 1.37E−02). Twelve of 13 enzymes assigned to this pathway in KEGG were unique to genomes in ecotype 1, including alpha‐ and beta‐galactosidase, alpha‐glucosidase, and maltodextrin glucosidase (Figure 2c). Notably, beta‐galactosidase, alpha‐galactosidase, and alpha‐glucosidase are enzymes that may enhance virulence by degrading components of vaginal mucus (Wiggins et al., 2001). Several enzymes involved in galactose metabolism were also assigned to pathways for sphingolipid metabolism, glycerolipid metabolism, glycosaminoglycan degradation, and glycosphingolipid biosynthesis. The pathway for “pentose and glucuronate interconversions” was enriched in ecotype 1 (OR = 2.86, *q* = 1.04E−03). Most of the enzymes assigned to this pathway (7/8) were uniquely present in genomes of ecotype 1 and were primarily involved in xylose, ribose, and arabinose metabolism. These findings suggest an enhanced ability among strains within this group to utilize galactose and 5‐carbon sugars as part of the cell's central metabolism, potentially providing a competitive advantage while co‐colonizing with other lactic acid bacteria.

In addition to the biochemical pathways mentioned above, ecotype 1 was also enriched for several protein families, including several ABC transporters and permeases that could serve a variety of functions involving transport of molecules across cell membranes. One of the most intriguing findings was the presence of a single protein family (clust_1151) annotated as zeta toxin, present in all isolates of ecotype 1 and absent from both ecotype 2 (*q* = 1.95E−02) and ecotype 3. Zeta toxins are highly homologous to PezT, the toxin component of the PezAT toxin–antitoxin (TA) system, which has been shown to kill bacteria by inhibiting peptidoglycan biosynthesis (Mutschler, Gebhardt, Shoeman, & Meinhart, 2011). Mutschler et al. described it as a “potent Achilles’ heel for microbes” and showed that partial autolysis caused by PezT in subpopulations of *Streptococcus pneumoniae* could favor biofilm formation. Zeta toxin might perform a similar function in strains of *G. vaginalis*, although this remains to be demonstrated experimentally.

### 3.8 Potential phage resistance mechanisms differ among lineages, with implications for *G. vaginalis* diversification

Throughout our gene enrichment analyses at different depths in the phylogeny, we noted an interesting pattern of enrichment for possible phage resistance mechanisms across clades and subclades. For example, for a monophyletic subset of lineages in ecotype 1 that we have designated 1B, we found two genes that encode for abortive infection proteins AbiGI and AbiGII that were not present in the remaining lineages of ecotype 1. These genes putatively comprise a type IV toxin–antitoxin system that confers resistance to some lactococcal bacteriophages (Tangney and Fitzgerald, 2002). Another gene encoding a “phage‐associated protein” was greatly elevated in all of strains of ecotype 1 relative to ecotypes 2 and 3 (OR = 9.84, *q *=* *1.37E−02). Ecotype 3 was enriched for three genes that encode proteins involved in a type I restriction‐modification system and uniquely possesses a WhiB‐like transcription regulator that could potentially be involved in resistance to mycobacteriophages (Rybniker et al., 2010). Restriction‐modification systems have been well described as protective of the cell against foreign DNA. These findings hint at different mechanisms of phage resistance that could influence how particular strains of *G. vaginalis* interact with other members of any given vaginal microbial community.

## 4 DISCUSSION

The serpentine history of the taxonomic classification of *G. vaginalis* is telling of the remarkable diversity within this species. *G. vaginalis* was renamed serially until the mid‐1990s, when researchers first noted the close relationship between strain ATCC 14018 (originally isolated by Gardner and Dukes) and *Bifidobacterium* spp. based on 16S rRNA gene sequences (Embley & Stackebrandt, 1994; Leblond‐Bourget, Philippe, Mangin, & Decaris, 1996). LeBlond‐Bourget et al. reasoned that although the similarity between *Gardnerella* and *Bifidobacterium* sequences was great enough to warrant assignment to the same genus, weak DNA–DNA hybridization and nonoverlapping ranges in GC content supported keeping the two genera separate. The close phylogenetic relationship of these two genera was reinforced by the discovery in *G. vaginalis* of the gene for fructose‐6‐phosphate phosphoketolase (F6PPK), an enzyme that was previously thought to occur only in *Bifidobacterium* spp. (Gavini, Van Esbroeck, Touzel, & Fourment, 1996). Thus, 40 years after its initial discovery, *Gardnerella* found its place within the family Bifidobacteriales.

Our findings indicate that most of the core genome of *Gardnerella* overlaps with the core genome of six *Bifidobacterium* spp., but it has a large accessory genome including many protein families that are unique to *Gardnerella. G. vaginalis* genomes are substantially smaller and have a significantly lower GC content when compared to *Bifidobacterium*, a pattern that has previously been observed in many bacterial symbionts (McCutcheon & Moran, 2012). Despite having up to 95% similarity in 16S rRNA gene sequences, major differences in the genomic composition of these two genera probably reflect a deep evolutionary split and broadly different ecology. It is especially intriguing in our comparison of *G. vaginalis* and *Bifidobacterium* that the former presents a much smaller genome size on average, and the absence of what could be considered essential pathways. These pathways, which are severely underrepresented in *G. vaginalis*, are involved in starch and sucrose metabolism, as well as galactose metabolism. Yet, *G. vaginalis* presents an enrichment of protein encoding genes responsible for multiple sugar transport into cells, a pattern that together is difficult to explain. It is possible that, given that these species do not exist in isolation and are found in co‐existence with multiple species of a community an within a living host, either the host or other community members could supply the products necessary for the subsistence of *G. vaginalis*.

The clades defined in this work have an operational purpose only, as they serve as the framework to ask whether ecotypes can be identified via gene enrichment analysis. Nevertheless, we see the need to put these definitions in a larger context and compare them to already published attempts to classify *G. vaginalis* lineages. Clades 1 and 2 defined here include isolates reported for clades 1 and 2 in Ahmed et al. (2012) and clades C and B (respectively) reported by Schellenberg et al. (2017). Our clade 3 corresponded to clades 3 and 4 in Ahmed et al. (2012) and clades D and A (respectively) reported by Schellenberg et al. (2017). The reason to consider previously reported clades 3 and 4 (as in Ahmed et al.) in a single group is purely operational, and we attempt not to bias our gene enrichment analysis by comparing clades with much fewer lineages. As the number of genomes in these groups increases, it will be easy to perform more refined analyses comparing the potential for functional diversification in these clades.

Despite the overall agreement in clade assignment with previous work, there are noteworthy differences to mention. Our approximation to reconstruct the phylogenetic relationships among *G. vaginalis* strains provides strong support all along different hierarchical levels of organization. The strains JCP8481A and JCP8481B that get assigned to clade A in Schellenberg et al. (2017) (4 in Ahmed et al., 2012) seem to be incorrectly assigned. In our reconstruction, these strains are confidently assigned to clade 3A (see Figure 2a) (clade 3 in Ahmed et al., 2012). This difference is particularly important given that strains JCP8481A and JCP8481B lack sialidases and when clades 3A and 3B (3 and 4 in Ahmed et al., 2012) are compared, no significant differences in the content of sialidases can be found.

Our analysis of 35 isolates of *G. vaginalis* supports the existence of three major ecotypes based on the phylogenetic structure of their core and accessory genes and the cohesiveness in functional gene composition within ecotypes. These ecotypes likely evolved in response to different selective pressures imposed by differences among microbial communities and hosts, although these patterns might have arisen through neutral turnover of genes in strains evolving as segregated populations.

It is important to emphasize that the phylogenetic relationships of *G. vaginalis* inferred in this work are consistent with the findings of Ahmed et al. (2012) based on a smaller set of 17 genomes and only looking at gene content in a model‐free comparison, as well as a recent study that included four *G. vaginalis* isolates from the bladder genome (Malki et al., 2016). Ahmed et al. remarked that the genomic diversity among strains was great enough to warrant designation as four separate species. We recapitulated similar groupings of strains using a total‐evidence phylogenetic reconstruction of both the core and accessory genes, but our gene enrichment analysis did not reveal any significant differences in functional genes between the clades previously recognized by Ahmed et al. as clades B‐3 and B‐4.

Our study provides insight into the potential ecological differences among lineages of *G. vaginalis* and supports an emerging view that particular ecotypes may possess greater virulence potential while others might be relatively benign. We found that the genomes of isolates in ecotype 1 uniquely encode several glycosidases (e.g., galactosidases, glucosidases, and fucosidases) and have expanded capabilities for galactose and pentose sugar metabolism. The most notable feature of isolates in ecotype 2 is the possession of at least two distinct genes encoding sialidase (also a type of glycosidase). This echoes an earlier report of multiple sialidase alleles that were predicted to be functionally similar (Santiago et al., 2011); however, strains in ecotype 2 were not included in that study. Interestingly, our results indicate that a majority of genomes in ecotype 3 lack genes for any of these enzymes. This point is especially noteworthy considering the observations of Balashov et al. that strains that we identified as lineages showing an underrepresentation of sialidases were more prevalent among both healthy and BV‐positive subjects, and strains that we identified as belonging to clade/ecotype 1 were positively associated with symptomatic BV. Glycosidases represent a large family of enzymes that are capable of degrading large, glycosylated mucin proteins (Wiggins et al., 2001). Activity of such enzymes may increase susceptibility to infection by thinning the protective layer of vaginal mucus (Briselden, Moncla, Stevens, & Hillier, 1992; Cauci et al., 1993). Moncla et al. recently demonstrated that enzymatic activities of four glycosidases present in *G. vaginalis*—sialidase, alpha‐galactosidase, beta‐galactosidase and alpha‐fucosidase—were positively associated with BV diagnosed based on Nugent scores (Moncla et al., 2015). Clade membership of strains was not assessed in the study by Moncla et al., but taken together with our findings, we could reasonably anticipate that strains in some clades might have an enhanced ability to degrade components of vaginal mucus, thus enabling direct contact with epithelial cell surfaces (Wiggins et al., 2001). Alpha‐fucosidase has also been suggested as a virulence factor, although Moncla et al. (2015) could not demonstrate a significant relationship between alpha‐fucosidase activity and BV. These results are particularly interesting in view of the marginal difference in expression that have been identified between BV and non‐BV strains in vitro for vaginolysin (Castro et al., 2015).

Recent work has shown that genomes of *G. vaginalis* isolated from bladder, vagina, and endometrium carry more than 400 annotated prophage sequences (Hardy et al., 2017). Additional evidence suggests that prophage acquisition occurs regularly and likely shapes the genetic diversity in the species. Our findings that ecotypes present differential enrichment of protein encoding genes associated with phage protection (type I restriction‐modification system, WhiB‐like transcription factor, among others) further suggest that selective pressure by phages is a main driver for the diversification of ecotypes in *G. vaginalis*. If this were the case, given the observation that prophage acquisition seems to be an ongoing process, we propose that further diversification of the species could be driven by the phage community infecting *G. vaginalis*, although direct evidence for such phage is lacking.

Recognition of multiple clades as distinct ecological entities can significantly improve our understanding of the role of *G. vaginalis* in health and disease. Moreover, we think it is critical to embrace the within‐species diversity of *G. vaginalis* to gain meaningful insight into its ecology. Our study provides preliminary evidence for ecotypes of *G. vaginalis*, but the high degree of diversity even within these groups suggests we have only scratched the surface. Future studies should seek to sample and sequence a greater variety and number of isolates, including a larger number of isolates from healthy women, adolescent girls and postmenopausal women, and women from many geographical regions. To develop further support for ecological distinctness among clades of *G. vaginalis*, studies should measure realized genetic potential with analyses of gene expression, as well as test the effects of interspecies interactions of each ecotype with other bacteria.
