# Supplementary figures and images for: Focusing the diversity of Gardnerella vaginalis through the lens of ecotypes
Source: Evol Appl. 2017 Nov 16;11(3):312–24. doi: 10.1111/eva.12555 (PMC5881158; doi:10.1111/eva.12555)

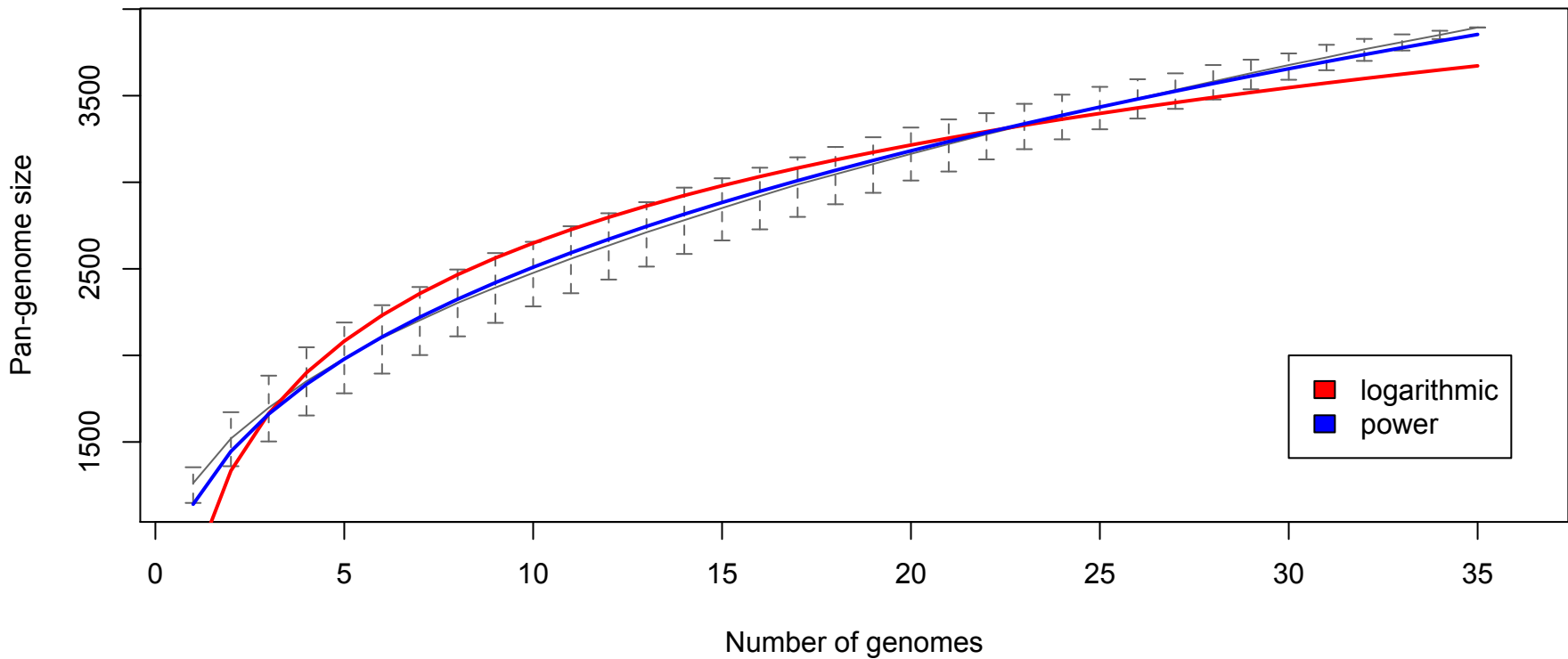

Supplement: Supplementary file 1 [file EVA-11-312-s001.pdf]

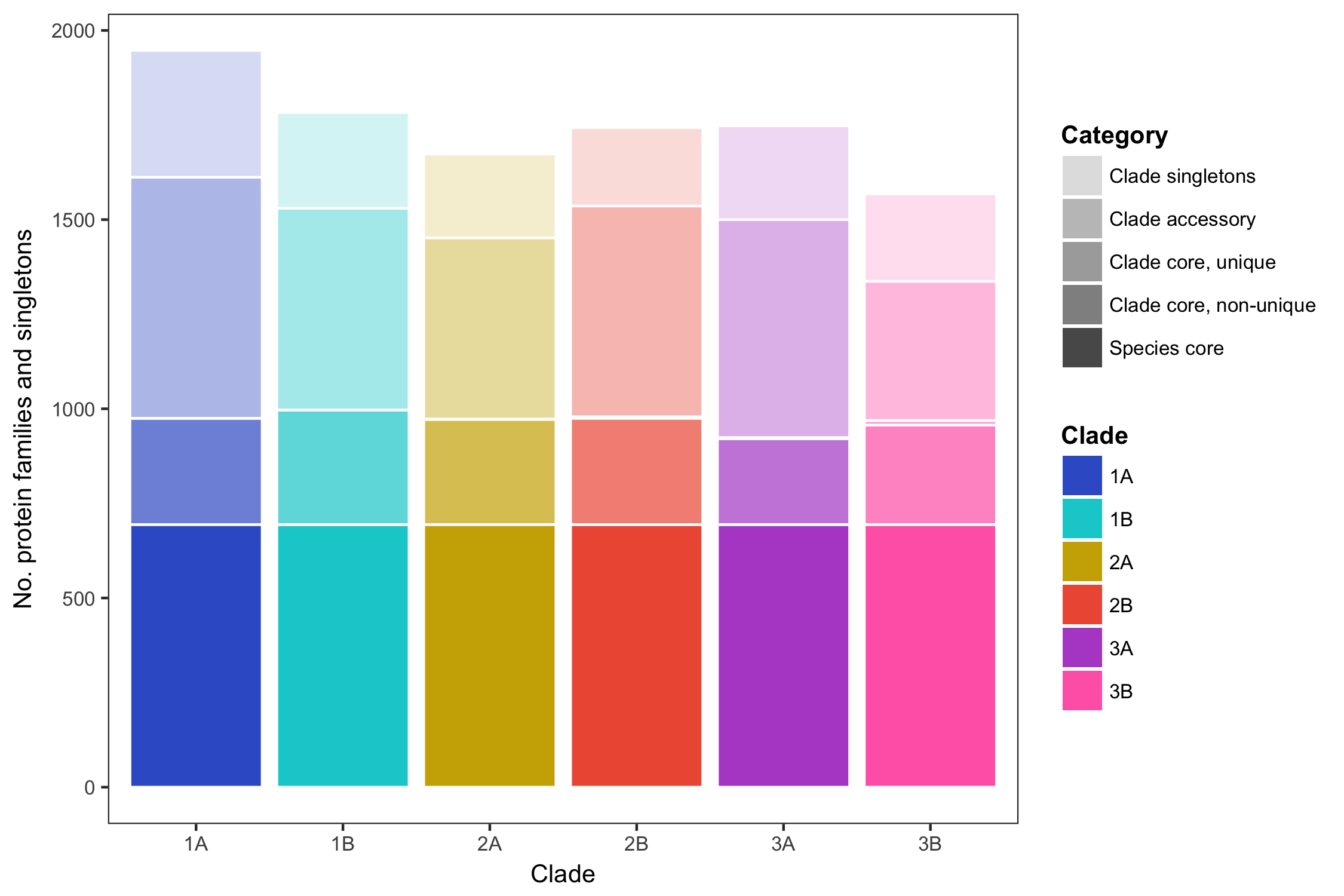

Supplement: Supplementary file 2 [file EVA-11-312-s002.png]
