# Supplementary material for: Focusing the diversity of Gardnerella vaginalis through the lens of ecotypes
Source: Evol Appl. 2017 Nov 16;11(3):312–24. doi: 10.1111/eva.12555 (PMC5881158; doi:10.1111/eva.12555)
Supplement: Supplementary file 3 [file EVA-11-312-s003.pdf]

Table S1. Genomic and clinical characteristics of 20 *Bifidobacterium* spp. strains.

Strain	GenBank accession(s)	Genomic characteristics[a]					Clinical and phenotypic characteristics[b]	
		Size (Mb)	Contigs	Plasmids	GC%	CDS	Source	Specific Comments
Bifidobacterium adolescentis L2-32	AAXD00000000	2.389	35	0	59.2	2,031	GI tract	Infant feces
Bifidobacterium animalis subsp. lactis B420	CP003497.1	1.939	1	0	60.5	1,604	Unknown	NR
Bifidobacterium animalis subsp. lactis Bi-07	CP003498.1	1.939	1	0	60.5	1,601	Unknown	NR
Bifidobacterium animalis subsp. lactis V9	CP001892	1.944	1	0	60.5	1,613	GI tract	Infant feces
Bifidobacterium bifidum BGN4	CP001361.1	2.224	1	0	62.6	1,828	GI tract	Feces
Bifidobacterium breve 12L	CP006711.1	2.245	1	0	58.9	1,907	Mammary gland	Human milk
Bifidobacterium breve 689b	CP006715.1	2.332	1	0	58.7	1,991	GI tract	Infant feces
Bifidobacterium breve ACS-071-V-Sch8b	CP002743	2.327	1	0	58.7	1,972	Urogenital tract	NR
Bifidobacterium breve JCM 7017	CP006712.1	2.289	1	0	58.7	1,944	GI tract	Infant feces
Bifidobacterium breve JCM 7019	CP006713.1	2.359	1	0	58.6	2,073	GI tract	Adult feces
Bifidobacterium breve JCP7499	AWSX00000000	2.367	91	0	58.6	2,073	Urogenital tract	Vagina
Bifidobacterium breve NCFB 2258	CP006714.1	2.316	1	0	58.7	1,978	GI tract	Infant feces
Bifidobacterium breve S27	CP006716.1	2.294	1	0	58.7	1,948	GI tract	Infant feces
Bifidobacterium breve UCC2003	CP000303	2.423	1	0	58.7	2,071	GI tract	Nursing infant feces
Bifidobacterium longum NCC2705	AE014295, AF540971	2.26	1	1	60.1	1,868	GI tract	Infant feces
Bifidobacterium longum subsp. infantis 157F	AP010890, AP010891, AP010892	2.409	1	2	60.1	2,058	GI tract	NR
Bifidobacterium longum subsp. longum BBMN68	CP002286	2.266	1	0	59.9	1,882	GI tract	Long-lived adult male
Bifidobacterium longum subsp. longum F8	FP929034	2.385	1	0	59.6	1,997	GI tract	NR
Bifidobacterium longum subsp. longum JCM 1217	AP010888	2.385	1	0	60.3	1,998	GI tract	NR
Bifidobacterium thermophilum RBL67	CP004346.1	2.292	1	0	60.1	1,839	GI tract	Infant feces

[a] Genomes were downloaded from the PATRIC database (ftp://ftp.patricbrc.org/patric2/) in March 2015. CDS = coding DNA sequence.

[b] Gathered from information available in PATRIC. NR = not reported.
