# Supplementary material for: Focusing the diversity of Gardnerella vaginalis through the lens of ecotypes
Source: Evol Appl. 2017 Nov 16;11(3):312–24. doi: 10.1111/eva.12555 (PMC5881158; doi:10.1111/eva.12555)
Supplement: Supplementary file 7 [file EVA-11-312-s007.docx]

# Supplementary Information

Figure S1. Number of protein families in the pan-genome of *G. vaginalis*. Accumulation rarefaction curves across 35 *G. vaginalis* strains were calculated based on the presence or absence of protein families and singleton coding genes using the “specaccum” function in the vegan R package and were estimated by bootstrapping 100 permutations of randomized sample order. The curves for all the genomes analyzed in this study (gray and errors in grey dotted lines) and fitted expectations for a power model of the form $cg=b*{ng}^{a}$ (blue line) where *cg* is number of new genes and *ng* the number of genomes (best fit) and a logistic model (red line).

Figure S2. Core, accessory and unique protein family counts within each clade of *G. vaginalis*. Genomes are grouped and colored by putative clades that were determined from the phylogeny in Figure 2. Protein family counts are plotted as stacked bars, with the density of shading corresponding to, in order from darkest to lightest: number of core protein families among the entire species; number of protein families core but not unique to each clade; number of protein families core and unique to each clade; number of protein families present in two or more genomes within each clade; and number of proteins that are present in a single genome within a clade. The class of proteins that are core and unique to each clade are present in a minority of cases and their representation is minimal.

Table S1. Genomic and clinical characteristics of 20 *Bifidobacterium* spp. strains.

**Supplementary Data Files**

File S1. Comprehensive results of protein family enrichment analysis on 35 strains of *G. vaginalis* strains and 20 strains of *Bifidobacterium* spp. (XLSX file)

File S2. Comprehensive results of protein family enrichment analysis on ecotype 3 vs ecotype 1/2 isolates of *G. vaginalis* grouped by phylogenetic clade. (XLSX file)

File S3. Comprehensive results of protein family enrichment analysis on ecotype 1 vs ecotype 2 isolates of *G. vaginalis* grouped by phylogenetic clade. (XLSX file)
